# Fertility Awareness and Parenthood Intentions Among Physicians in Jazan, Saudi Arabia: A Cross-Sectional Study

**DOI:** 10.7759/cureus.89148

**Published:** 2025-07-31

**Authors:** Tahani Shar, Fatima Bakheit

**Affiliations:** 1 Department of Family Medicine, Jazan Health Cluster, Ministry of Health, Jazan, SAU; 2 Department of Community Medicine, Jazan Health Cluster, Ministry of Health, Jazan, SAU

**Keywords:** attitude, awareness, : fertility, healthcare workers, parenthood, saudi arabia

## Abstract

Introduction

Many physicians face delays in childbearing due to demanding medical training and work environments, leading to high rates of infertility. In Saudi Arabia, societal shifts such as increased female education and professional participation further influence reproductive behavior. This study aimed to assess fertility awareness, intentions, and attitudes toward parenthood among physicians in the Jazan region of Saudi Arabia.

Methods

A descriptive cross-sectional study was conducted among family and general physicians working in eight healthcare sectors in Jazan. A stratified random sampling method with proportional allocation was used, targeting a minimum sample size of 284. Participants were invited via email to complete a self-administered questionnaire adapted from the literature, covering fertility awareness, childbearing intentions, influencing factors, and demographic characteristics. Statistical significance was set at *p* < 0.05.

Results

The median age of participants was 36 years; 55.4% were male and 44.6% were female. Most had children, and 73.5% intended to have (more) children. Peak female fertility was identified as 20-24 years, with 25-29 years being the age of slight fertility decline. The average willingness to undergo in vitro fertilization (IVF) was 7.8%. Reported obstacles included education, personal interests, and career advancement.

Conclusion

Physicians in Jazan demonstrated moderate fertility awareness, with notable gaps in knowledge of age-related fertility decline and IVF success rates. Parenthood intentions were influenced more by relationship and lifestyle factors than by medical knowledge. Targeted fertility education and workplace support are needed to empower physicians to make informed reproductive decisions aligned with their professional and personal goals.

## Introduction

In recent years, the global fertility rate has shown a steady decline. In 2021, the worldwide fertility rate was estimated at 2.23 (2.09-2.38) and is projected to drop further to 1.83 (1.59-2.08) by 2050. However, fertility rates in North Africa and the Middle East were slightly higher at 2.53 (2.33-2.76) in 2021, with projections indicating a decrease to 1.94 (1.62-2.28) by 2050. In Saudi Arabia specifically, the fertility rate in 2021 was 1.44 (1.24-1.68) and is expected to decline to 1.09 (0.80-1.39) by 2050 [1]. Deciding when to become a parent involves complex considerations, including personal aspirations and career goals, balanced against the biological limits of fertility [2]. Studies suggest that delayed parenthood is influenced by insufficient knowledge about fertility and a strong belief in the success of assisted reproductive technologies [3].

Female physicians, in particular, often delay childbearing due to the demands of medical education and career development. On average, first pregnancies occur at 30.4 years, with infertility typically diagnosed around 33.7 years of age [4]. Among those who tried to conceive, 24.1% reported infertility diagnoses, and some were found to have diminished ovarian reserve, reflecting the consequences of delayed reproduction [4]. Furthermore, 28.6% of surveyed female physicians stated they would have attempted conception earlier had they been more informed about fertility decline. About 7% would have considered fertility preservation options such as cryopreservation [4].

Professional challenges, including inadequate support for pregnancy and breastfeeding, along with long training hours and workloads, contribute significantly to postponing parenthood among women physicians. Increasing fertility awareness could encourage earlier family planning or promote advocacy for institutional reforms [5].

Improving fertility education during medical training and throughout physicians' careers is essential to support informed reproductive decisions [6]. Providing better workplace support and access to fertility preservation services may help physicians align their family planning with personal values and professional aspirations [6]. Research also indicates that perceptions of workplace support can influence family planning decisions, physicians who became pregnant during training felt less supported than those who delayed pregnancy until after training [4].

International studies have examined fertility awareness among various populations. For example, in Sweden, students often overestimated women’s fertility but had generally realistic views of the most fertile period in life [7]. Among Chinese university students, 66% overestimated the success rate of assisted reproductive technologies, and 92% underestimated the impact of age on fertility. Interestingly, many were less inclined to pursue parenthood, more aware of infertility, and less troubled by the idea [8]. In contrast, medical students in Sweden, Belgium, and Greece demonstrated satisfactory fertility knowledge [9].

Despite global research on fertility awareness, there is limited data from Saudi Arabia. One study at King Saud University in Riyadh found that education had a significant impact on fertility-related attitudes, including age at marriage, preferred family size, number of children, and contraceptive use. High fertility remains a prominent cultural and historical value in Saudi society [10].

In a country like Saudi Arabia, where traditional values promote high fertility [10], changing social conditions are beginning to reshape reproductive choices. Increased educational attainment among women has led to delays in marriage and childbirth. Moreover, the high-pressure environment of the medical profession may further influence these decisions [11]. Given these changes, fertility awareness is becoming an increasingly important issue. Accordingly, this study aims to assess fertility awareness, intentions, and attitudes toward parenthood among physicians in Jazan, Saudi Arabia.

## Materials and methods

Study design, population, and area

This descriptive cross-sectional study was conducted at primary health care (PHC) centers in the Jazan region, located in the southwestern part of Saudi Arabia along the Red Sea coast. The region hosts 554 physicians working across 165 PHC centers, which are organized into eight sectors under the Ministry of Health.

The study targeted all family and general physicians working in these PHC centers, irrespective of their professional level. It excluded physicians from specialties other than family medicine, healthcare professionals outside of family medicine, family physicians employed by security or military forces, and undergraduate medical students.

Sample size calculation

The sample size was calculated using the Raosoft sample size calculator [12]. With a margin of error of 5%, a 95% confidence interval, a 50% proportion, and a population of 554 physicians, the required sample size was 228. Accounting for a 20% non-response rate, the final sample size was increased to 284 participants. Proportional allocation was used to determine the sample size for each stratum within the population, as calculated using the following formula [13]:

$$n_h = \left( \frac{N_h}{N} \right) \times n$$

Where:

$n_h$: Sample size for stratum $h$

$N_h$: Population size of stratum $h$

$N$: Total population size

$n$: Total sample size

A detailed description of the sample size estimation for each sector is provided in Appendix 1.

Sampling technique

A stratified random sampling method was employed. A list of physicians was obtained for each sector, and random numbers were generated based on the calculated sample size for each sector. Selected physicians were contacted via email, which included an information sheet outlining the purpose and nature of the study, as well as a link to a Google Form for data collection. Two reminders were sent, with a one-week interval between them.

Data collection method

Data were collected using a self-administered questionnaire adapted from Lampic C et al. (2006) [7], with permission obtained from the original publisher (Appendix 2). The questionnaire, in English, consisted of five domains (Appendix 3). Its face validity and reliability were deemed satisfactory. The first domain assessed demographic characteristics such as age, gender, years of practice, specialty, and marital status.

The second domain, regarding intentions to have children, included one yes/no question on plans to have children and three open-ended questions on the desired number of children, the preferred age at parenthood (total of 4 items), and potential obstacles to achieving fertility goals.

The third domain assessed awareness of fertility issues and contained 9 items focusing on women's fertility, odds of pregnancy, and infertility. These items were assessed using open-ended questions.

The fourth domain used a 0-10 point scale to assess the importance of having children (1 item) and behavioral intentions in the event of infertility (3 items).

The fifth domain assessed how important each factor was in the decision to pursue parenthood, using a six-point Likert scale with responses ranging from “unimportant” to “very important.”

Statistical analysis

Data were gathered in Microsoft Excel, cleaned, and analyzed using SPSS version 26. The normality of continuous variables was assessed using histograms and the Kolmogorov-Smirnov test. Descriptive statistics included frequencies and percentages for categorical variables, and median with IQR or mean (SD) for continuous variables. The awareness score was derived from Section 3, which included 9 open-ended items assessing knowledge of fertility issues such as the age of peak fertility, age-related fertility decline, and IVF success rates. Each correct response was scored as one point, while incorrect or missing responses were scored as zero. The total possible score ranged from 0 to 9. Participants who answered 0 items correctly were classified as having low awareness; those who answered 1-2 correctly had moderate awareness; and those who answered 3-4 correctly were classified as having high awareness. No participants answered more than 5 of the 9 awareness-related items correctly. Pearson's Chi-squared and Fisher's exact tests were performed to identify the association between knowledge of fertility issues and gender. Statistical significance was set at p < 0.05.

Ethical considerations

Ethical approval was obtained from the Jazan Ethics Committee, Jazan Health Cluster, Saudi Arabia (No: 2485). All participants were provided with an information sheet detailing the purpose and nature of the study. Participants were informed that their involvement was voluntary and that they could withdraw from the study without penalty. Strict confidentiality was maintained throughout the study, and all collected data were securely stored.

## Results

Two hundred thirteen healthcare professionals participated in the study, with a median age of 36 years (IQR: 32-43). The final sample size was 213 participants, slightly below the calculated target of 228 due to difficulty in recruiting physicians and a low response rate. More than half were male (118/213, 55.4%). A total of 80/213 (37.6%) were family medicine specialists or consultants, 77/213 (36.2%) were general practitioners, 44/213 (20.7%) were family medicine residents, and 12/213 (5.6%) belonged to other specialties.

Among the participants, 64/213 (30.1%) had 6-10 years of professional experience, 62/213 (29.1%) had 1-5 years, 55/213 (25.8%) had 11-15 years, and 32/213 (15.0%) had more than 15 years of experience. Regarding fertility education, 94/213 (44.1%) perceived themselves as educated, and 80/213 (37.6%) as somewhat educated. Additionally, 24/213 (11.3%) reported being very educated, 14/213 (6.6%) not very educated, and 1/213 (0.5%) not at all educated. Doctors or gynecologists were the most commonly reported source of fertility information (153/213, 71.8%), followed by schools (78/213, 36.6%), media (57/213, 26.8%), family (23/213, 10.8%), friends (22/213, 10.3%), medical websites (4/213, 1.9%), medical references (3/213, 1.4%), and training programs (2/213, 0.9%) (Table 1).

**Table 1:** Demographic, professional, and fertility-related attributes of participants. ¹ n (%); median (IQR) ² Percentages are calculated based on the number of valid responses (excluding missing data). * Multiple-choice question

Characteristics	N = 213¹,²
Gender	
Female	95 (44.6%)
Male	118 (55.4%)
Age	36 (32, 43)
Specialty	
Family medicine (specialist, consultant)	80 (37.6%)
Family medicine resident	44 (20.7%)
General practitioner	77 (36.2%)
Others	12 (5.6%)
Years of experience	
1-5 years	62 (29.1%)
6-10 years	64 (30.1%)
11-15 years	55 (25.8%)
More than 15 years	32 (15.0%)
Perceived level of education regarding fertility	
Not at all educated	1 (0.5%)
Not very educated	14 (6.6%)
Somewhat educated	80 (37.6%)
Educated	94 (44.1%)
Very educated	24 (11.3%)
Source of information regarding fertility*	
Doctor/gynecologist	153 (71.8%)
School	78 (36.6%)
Media	57 (26.8%)
Family	23 (10.8%)
Friends	22 (10.3%)
Medical websites	4 (1.9%)
Medical references	3 (1.4%)
Training programs	2 (0.9%)

A total of 163/213 (76.5%) reported having children, while 50/213 (23.5%) did not. Among those who did not have children, 36/49 (73.5%) planned to have children in the future. Furthermore, 101/204 participants (49.5%) preferred having 4 to 6 children, while 78/204 (38.2%) preferred 1 to 3 children.

Regarding the desired age to have the first child, 77/123 participants (62.6%) preferred between 20 and 30 years, while 46/123 (37.4%) preferred between 31 and 42 years. However, 25/123 (17.1%) had their first child between 20 and 25 years of age, 58/123 (39.7%) between 26 and 30 years, and 37/123 (25.3%) between 30 and 35 years. Regarding the desired age for having their last child, 141/202 participants (69.8%) chose between 32 and 45 years, 39/202 (19.3%) chose between 46 and 56 years, and 8/202 (3.9%) preferred between 20 and 30 years.

The average willingness to undergo in vitro fertilization was 7.8 (SD ±3.1), while the mean response for willingness to adopt was 4.0 (SD ±3.6), and for choosing not to have children was 2.11 (SD ±2.95) (Table 2).

**Table 2:** Overview of parenthood, childbearing intentions, and fertility preferences among study participants. ¹ n (%); mean ± SD ² Percentages are calculated based on the number of valid responses (excluding missing data).

Characteristic	N = 213¹,²
Have children	
No	50 (23.5%)
Yes	163 (76.5%)
If not, do you plan to have children?	
No	13 (26.5%)
Yes	36 (73.5%)
Unknown	164
Desired number of children	
0	16 (7.8%)
1-3	78 (38.2%)
4-6	101 (49.5%)
7-9	6 (2.9%)
10 or more	3 (1.5%)
Unknown	9
Desired age when having your first child	
20-30	77 (62.6%)
31-42	46 (37.4%)
Unknown	90
Actual age at which first child was had	
<20	16 (10.9%)
20-25	25 (17.1%)
26-30	58 (39.7%)
31-35	37 (25.3%)
36-40	10 (6.8%)
Unknown	67
Desired age when having your last child	
20-30	8 (3.9%)
31-45	141 (69.8%)
46-56	39 (19.3%)
57-60	5 (2.5%)
I don’t know	9 (4.5%)
Unknown	11
Willingness to undergo in vitro fertilization	7.8 ± 3.1
Willingness to adopt	4.0 ± 3.6
Willingness to choose not to have children	2.11 ± 2.95

Regarding the age at which women are most fertile, 56/207 participants (27.1%) correctly identified 20-24 years as the peak fertility age. Other responses included 15-19 years, selected by 40/207 participants (19.3%); 30-55 years, selected by 44/207 (32.4%); and 25-29 years, selected by 67/207 (21.3%). The difference between males and females was insignificant (p = 0.3).

Regarding the age at which there is a slight decrease in a woman's ability to become pregnant, 182/209 participants (87.1%) selected the range of 35-59 years, while 19/209 (9.1%) selected 30-34 years. Only 4/209 (1.9%) correctly selected the range of 25-29 years, and another 4/209 (1.9%) selected 15-24 years. The p-value was 0.078.

In response to the age at which there is a marked decrease in fertility, 125/211 participants (59.3%) selected 45-60 years, while 70/211 (33.2%) selected 40-44 years. Only 11/211 participants (5.2%) correctly chose 35-39 years. No significant gender difference was observed (p = 0.5).

When asked about the chance of pregnancy if a 25-year-old woman has unprotected intercourse during ovulation, 170/205 participants (82.9%) selected 50-100%. Only 14/205 participants (6.8%) selected the correct range of 30-39%. The p-value was 0.6.

For the chance of pregnancy in women aged 25-30, 92/206 participants (44.7%) selected 90-100%, followed by 53/206 (25.7%) choosing 80-89%. Only 32/206 (15.5%) chose the correct range of 70-79%. No significant gender difference was found (p = 0.2).

Regarding the chance of pregnancy for women aged 35-40, 90/207 participants (43.5%) estimated it at 70-100%; 44/207 (21.3%) chose 0-49%; another 44/207 (21.3%) correctly selected 50-59%; and 29/207 (14.0%) selected 60-69%. Males and females responded similarly (p = 0.8).

When asked about the percentage of couples in Saudi Arabia who are involuntarily childless, 50/182 (27.5%) selected the correct range of 10-19%. The p-value was 0.5.

For the average chance that a couple will conceive after one IVF cycle, 132/200 participants (66.0%) estimated 40-100%, while only 22/200 (11.0%) correctly selected 30-39%. The rest chose 20-29% (27/200, 13.5%) and 0-19% (19/200, 9.5%). There was no significant difference by gender (p = 0.3) (Table 3).

**Table 3:** Knowledge of fertility issues among participating healthcare providers. ¹ n (%); mean ± SD ² Percentages are calculated based on the number of valid responses (excluding missing data). ³ Pearson’s chi-squared test; Fisher’s exact test * Correct answer Adapted items from the original questionnaire by Lampic C et al. (2006) [7]. Reproduced with permission from the original publisher. This material is not distributed under a Creative Commons license.

Characteristic	Overall, N = 213¹,²	Female, N = 95¹,²	Male, N = 118¹,²	p-value³
Age at which women are most fertile				0.3
15-19	40 (19.3%)	12 (13.2%)	28 (24.1%)	
20-24*	56 (27.1%)	27 (29.7%)	29 (25.0%)	
25-29	44 (21.3%)	20 (21.9%)	24 (20.7%)	
30-55	67 (32.4%)	32 (35.2%)	35 (30.2%)	
Unknown	6	4	2	
Age at which there is a slight decrease in women's ability to become pregnant				0.078
15-24	4 (1.9%)	0 (0.0%)	4 (3.5%)	
25-29*	4 (1.9%)	2 (2.1%)	2 (1.7%)	
30-34	19 (9.1%)	5 (5.3%)	14 (12.2%)	
35-59	182 (87.1%)	87 (92.6%)	95 (82.6%)	
Unknown	4	1	3	
Age at which there is a marked decrease in women's ability to become pregnant				0.5
25-34	5 (2.4%)	1 (1.1%)	4 (3.4%)	
35-39*	11 (5.2%)	3 (3.2%)	8 (6.8%)	
40-44	70 (33.2%)	33 (35.1%)	37 (31.6%)	
45-60	125 (59.3%)	57 (60.6%)	68 (58.1%)	
Unknown	2	1	1	
A young woman, 25 years old, and a man have unprotected intercourse at the time of ovulation. The chance that she will become pregnant				0.6
0-29%	17 (8.3%)	6 (6.5%)	11 (9.7%)	
30-39%*	14 (6.8%)	5 (5.4%)	9 (7.9%)	
40-49%	4 (2.0%)	1 (1.1%)	3 (2.7%)	
50-100%	170 (82.9%)	80 (86.9%)	90 (79.6%)	
Unknown	8	3	5	
Chance: if she is 25-30 years old				0.2
0-69%	29 (14.1%)	9 (9.8%)	20 (17.5%)	
70-79%*	32 (15.5%)	12 (13.0%)	20 (17.5%)	
80-89%	53 (25.7%)	24 (26.1%)	29 (25.4%)	
90-100%	92 (44.7%)	47 (51.1%)	45 (39.5%)	
Unknown	7	3	4	
Chance: if she is 35-40 years old				0.8
0-49%	44 (21.3%)	17 (18.5%)	27 (23.5%)	
50-59%*	44 (21.3%)	19 (20.7%)	25 (21.7%)	
60-69%	29 (14.0%)	14 (15.2%)	15 (13.0%)	
70-100%	90 (43.5%)	42 (45.7%)	48 (41.7%)	
Unknown	6	3	3	
Percentage of couples in Saudi Arabia who are involuntarily childless				0.5
0-4%	15 (8.2%)	8 (10.8%)	7 (6.5%)	
5-9%	27 (14.8%)	9 (12.2%)	18 (16.7%)	
10-19%*	50 (27.5%)	18 (24.3%)	32 (29.6%)	
20-90%	90 (49.5%)	39 (52.7%)	51 (47.2%)	
Unknown	31	21	10	
The average chance that a couple who undergoes treatment with in vitro fertilization will have a child after one treatment				0.3
0-19%	19 (9.5%)	8 (9.0%)	11 (9.9%)	
20-29%	27 (13.5%)	8 (9.0%)	19 (17.1%)	
30-39%*	22 (11.0%)	9 (10.1%)	13 (11.7%)	
40-100%	132 (66.0%)	64 (71.9%)	68 (61.3%)	
Unknown	13	6	7	
Knowledge score (mean ± SD)	1.094 ± 0.98			

Approximately 16/213 (7.5%) had high knowledge of fertility issues, while the majority, 132/213 (62.0%), had moderate knowledge, and 65/213 (30.5%) had low knowledge.

**Figure 1:**
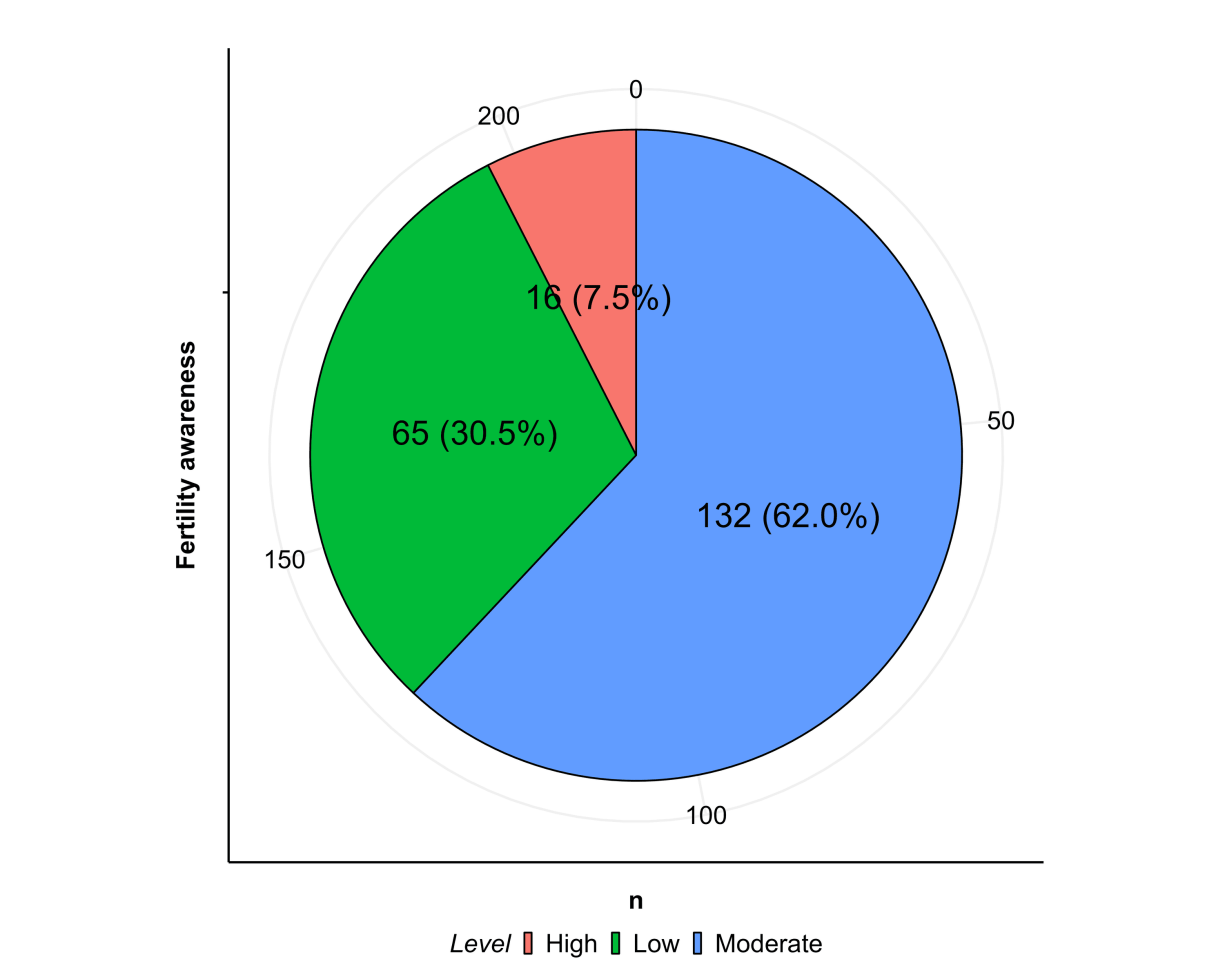
Knowledge levels of fertility awareness among study participants.

When stratified by gender, 8/118 (6.8%) males and 8/95 (8.4%) females had high knowledge levels; 76/118 (64.4%) males and 56/95 (58.9%) females had moderate knowledge; and 34/118 (28.8%) males and 31/95 (32.6%) females had low knowledge. No statistically significant difference was observed (p = 0.05) (Figure 1).

**Figure 2:**
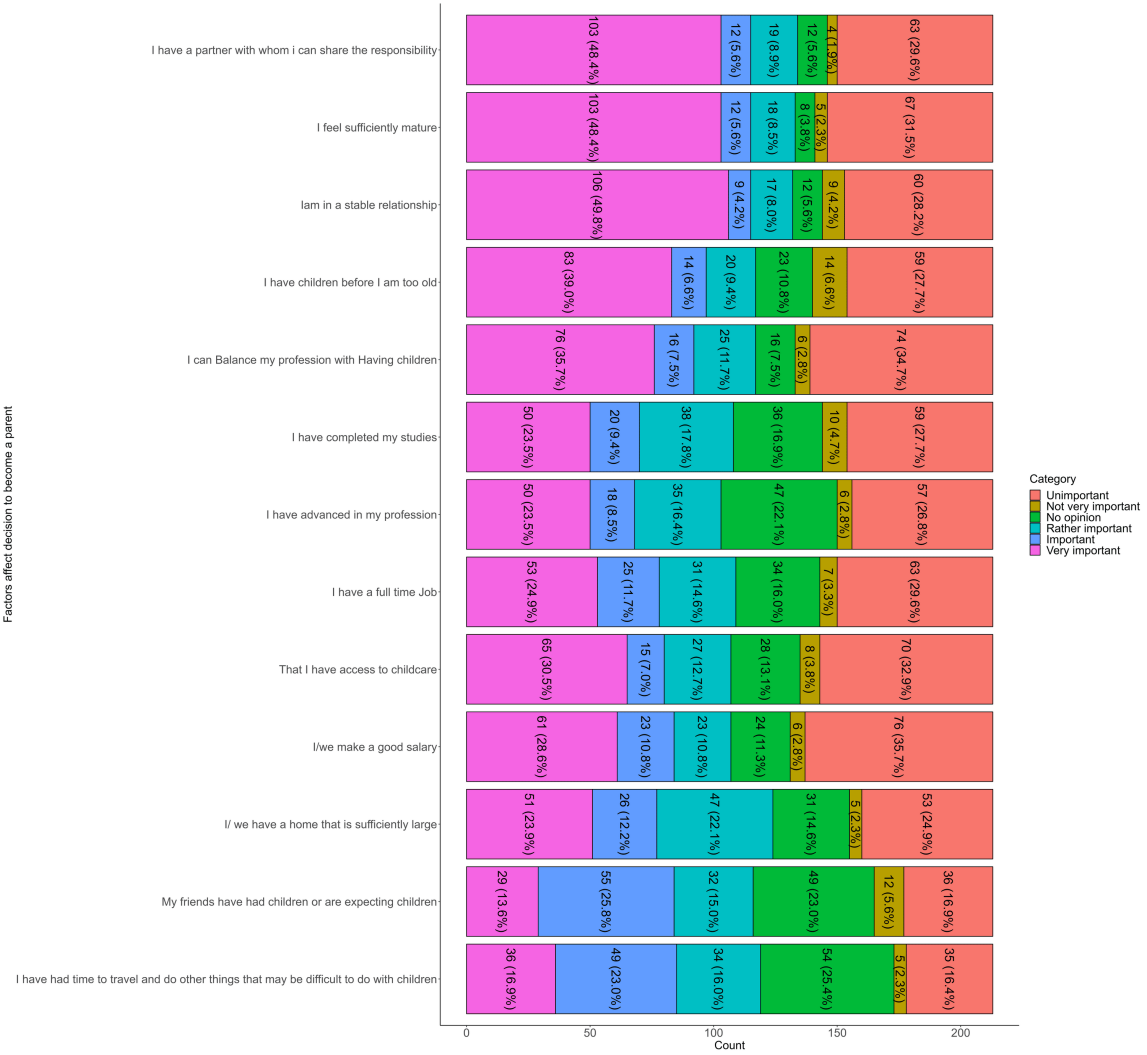
Perceived importance of factors influencing the decision of parenthood.

The most frequently cited factor affecting the decision to become a parent was having a partner to share responsibility, with 103/213 participants (48.4%) rating it as very important. Similarly, feeling sufficiently mature was rated very important by 103/213 (48.4%), and being in a stable relationship by 106/213 (49.8%). Avoiding late parenthood was considered very important by 83/213 (39.0%), while balancing profession with children was viewed as very important by 76/213 (35.7%). Completing one’s studies was rated very important by 50/213 (23.5%), and advancing in a profession by another 50/213 (23.5%). Having a full-time job was considered very important by 53/213 (24.9%), and access to childcare by 65/213 (30.5%). A good salary was very important for 61/213 (28.6%), and a sufficiently large home for 51/213 (23.9%). Social influence, such as friends having or expecting children, was less influential, with only 29/213 (13.6%) rating it as very important. Similarly, having time to travel or pursue other interests before having children was seen as very important by 36/213 (16.9%) (Figure 2).

**Figure 3:**
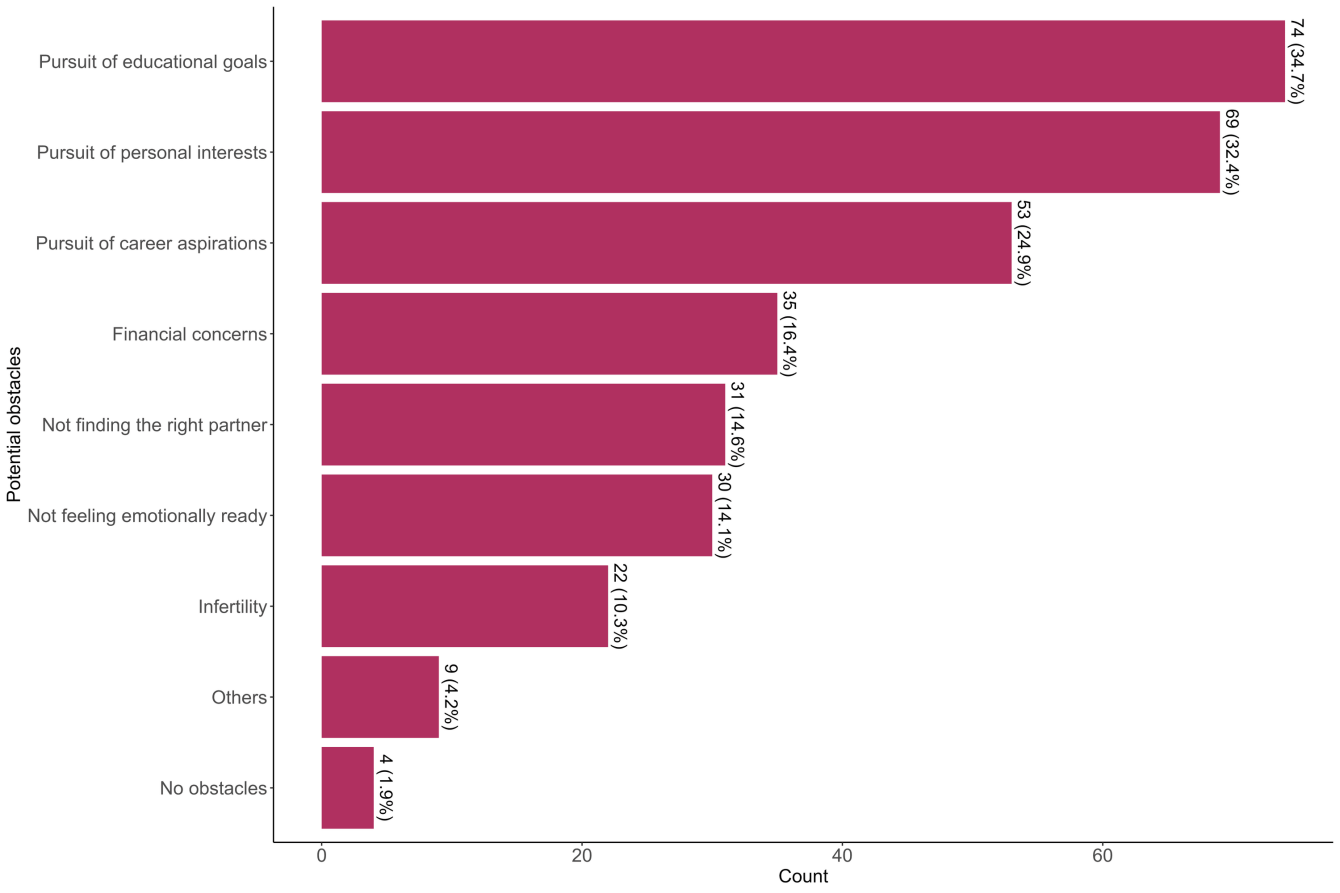
Potential obstacles to parenthood among participating healthcare professionals.

The most frequently reported potential obstacle to becoming a parent was the pursuit of educational goals, cited by 74/213 (34.7%), followed by the pursuit of personal interests, reported by 69/213 (32.4%), and career aspirations, reported by 53/213 (24.9%). Financial concerns were cited by 35/213 (16.4%), while 31/213 (14.6%) reported not finding the right partner, and 30/213 (14.1%) cited not feeling emotionally ready. Infertility was mentioned by 22/213 (10.3%), while other reasons were reported by 9/213 (4.2%). Only 4/213 (1.9%) stated they faced no obstacles to becoming a parent (Figure 3).

## Discussion

Due to the nature, length, and intensity of medical training, many physicians are choosing to delay childbearing, resulting in rising infertility rates [14]. These evolving factors make fertility a significant and compelling issue to explore. Therefore, this study examined fertility awareness, intentions, and attitudes toward parenthood among physicians in Jazan, Saudi Arabia.

Although 36/49 (73.5%) of the participants expressed intentions to have children in the future, the majority demonstrated low to moderate levels of fertility awareness. This level of awareness is comparable to findings from previous studies among medical trainees and obstetrics and gynecology (OB/GYN) residents in the USA [15,16]. However, our findings contradict a study conducted in Saudi Arabia among OB/GYN physicians, which reported higher awareness levels [17]. Fertility knowledge among general and family physicians is crucial, not only for their reproductive planning but also for providing accurate guidance to their patients, especially given the increasing rates of infertility and the growing demand for fertility treatments in Saudi Arabia [18].

The low awareness observed in our study may be attributed to the cultural sensitivity surrounding fertility, which is often considered a taboo subject. This leads to the accumulation of widespread misconceptions about reproductive health and fertility [19]. Notably, more than half of the physicians rated themselves as educated to very educated about fertility, yet an alarming proportion overestimated the age at which female fertility begins to decline, both slightly and markedly. This gap between perceived and actual knowledge is concerning, as it may result in misguided reproductive decisions based on inaccurate assumptions.

Approximately one-third of the participants, 56/207 (27.1%), correctly identified the age at which women are most fertile, a figure comparable to that reported in a previous Saudi study [20]. Interestingly, no significant gender differences were found in fertility awareness, which aligns with some Saudi studies but differs from others [21,22]. This inconsistency highlights the need for further qualitative research to better understand the influence of gender on fertility awareness.

Regarding attitudes toward parenthood, 163/213 (76.5%) of our participants already have children, and a similar proportion of those without children plan to have them. This finding aligns with a previous Saudi study and is higher than what has been reported in Danish research [20,23]. There was a notably low willingness among participants to remain childless, which may reflect cultural and religious influences, particularly in Muslim-majority societies like Saudi Arabia.

Half of the participants, 101/204 (49.5%), expressed a desire to have between four and six children, higher than the number desired by participants in South Korean and American studies [24]. Additionally, nearly 77/123 (62.6%) of participants preferred to have their first child between the ages of 20 and 30, while a slightly higher proportion, 141/202 (69.8%), wished to have their last child between the ages of 31 and 45. These reproductive plans raise concerns given the sharp decline in female fertility with age, with pregnancy success rates dropping to 29% at ages 38-40 and further declining to only 11% after the age of 40 [25]. Interestingly, many participants indicated a willingness to pursue in vitro fertilization (IVF) in the future. In 2018, Saudi Arabia had over 35 assisted reproductive technology (ART) centers performing approximately 20,000 IVF cycles annually [25]. This reflects growing confidence in medical advancements and reproductive technologies to support fertility.

The most frequently cited factor influencing the decision to become a parent was having a partner to share responsibility, with 103/213 (48.4%) rating this as very important, consistent with findings from a Korean study [26]. While men have traditionally held the role of primary decision-makers in reproductive matters within Islamic countries [27], the emphasis on shared responsibility suggests a growing trend toward more egalitarian parenting roles and joint decision-making among couples.

Likewise, feeling sufficiently mature and being in a stable relationship were rated as very important by 103/213 (48.4%) of the participants, supporting previous research that highlights emotional readiness and relationship stability as key determinants in the decision to have children [28]. Additionally, approximately one-third of participants considered having a full-time job (53/213, 24.9%), progressing in their profession (50/213, 23.5%), and earning a good salary (61/213, 28.6%) as very important in their decision to pursue parenthood. This aligns with findings from a prior study indicating that increased education and workforce participation among women are associated with lower fertility intentions and significantly influence reproductive decisions [29].

The most commonly reported potential barrier to becoming a parent was the pursuit of educational goals, mentioned by 74/213 participants (34.7%). This was followed by the pursuit of personal interests, reported by 69/213 (32.4%), and career advancement, reported by 53/213 (24.9%), all of which are well-documented in the literature [11,30]. Delaying childbearing until the completion of training or attainment of higher education may increase the risk of infertility among physicians and result in unmet reproductive goals. Although fewer participants cited financial concerns, this factor is well recognized in existing research [11].

This study offers several notable strengths. The stratified random sampling method strengthens the representativeness of findings among primary healthcare physicians in Jazan. As the first investigation in this region, it establishes important baseline insights into physicians' fertility awareness and attitudes toward parenthood. Using a validated questionnaire enabled a comprehensive assessment of reproductive decision-making factors. Furthermore, the strong participation rate and culturally contextualized results shed light on how professional and social dynamics shape fertility planning in this setting.

Several limitations warrant consideration. The study’s cross-sectional design precludes establishing causal relationships between fertility knowledge and reproductive choices. To reduce potential bias in self-reported measures, we used a validated questionnaire with established reliability. To minimize social desirability bias and encourage honest responses, data collection was conducted anonymously. Although subjective perceptions of knowledge may differ from objective understanding, clear instructions were provided to all participants to ensure consistency. While the geographically specific sample may limit generalizability, recruiting participants from diverse healthcare facilities across Jazan improved representativeness. Lastly, although the absence of qualitative components left some cultural and personal dimensions unexplored, the quantitative design allowed for broader participation and statistical comparison, which aligned with the study’s scope.

## Conclusions

Fertility and parenthood remain central concerns in the minds of physicians, influenced by a range of factors that shape their reproductive choices. Our study revealed a low to moderate level of fertility awareness among participants, despite a strong willingness to have children in the future. The most significant barriers to parenthood were educational pursuits, personal goals, and career advancement. It is essential to incorporate targeted fertility education into medical training and continuing professional development to address this knowledge gap. Doing so could enhance physicians’ understanding of reproductive health and support informed decision-making. Future qualitative research is recommended to gain deeper insights into physicians’ choices regarding parenthood. Additionally, longitudinal studies could explore the long-term impact of career demands on reproductive decisions. Institutional reforms, such as implementing flexible work schedules and offering childcare support, are also necessary to promote better work-life balance for medical professionals.

## Figures and Tables

**Figure 4:**
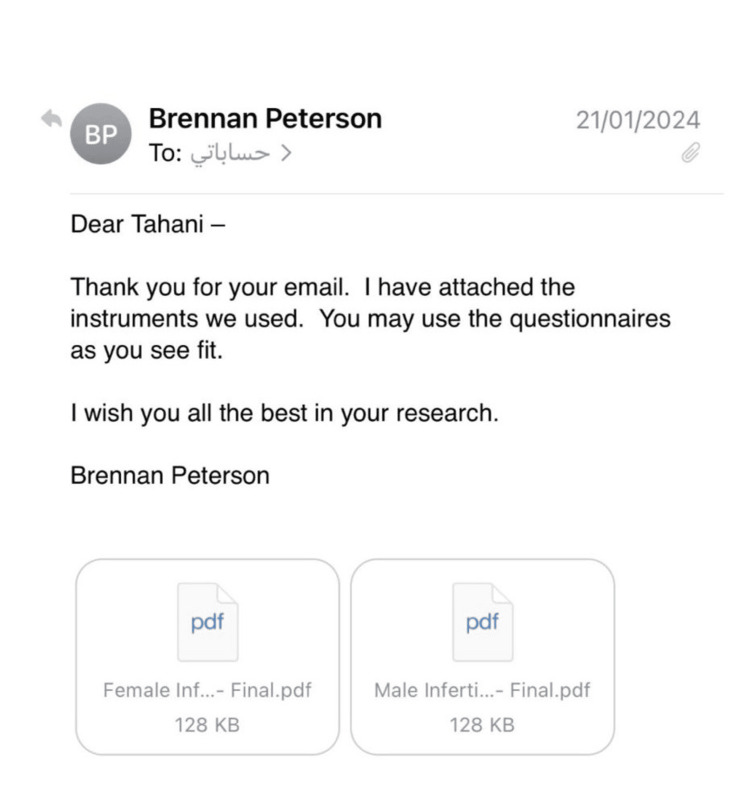
Permission obtained from the original publisher. Data were collected using a self-administered questionnaire adapted from Lampic C et al. (2006) [7], with permission obtained from the original publisher.

## Appendices

Appendix 1 

**Table 4:** Sample size estimation for each sector. $$n_h = \left( \frac{N_h}{N} \right) \times n$$ Where: $n_h$: Sample size for stratum $h$ $N_h$: Population size of stratum $h$ $N$: Total population size $n$: Total sample size

Sample size	Number of physicians	Healthcare sector
64	120	Middle
64	128	Central
64	128	Western
5	9	Fursan
18	35	Mountain
44	86	Northern
13	25	Southern
12	23	Bani Malek
284	554	Total

Appendix 2

Appendix 3 

**Table 5:** Questionnaire on fertility awareness and parenthood attitudes among physicians in Jazan, Saudi Arabia. The questionnaire, in English, consisted of four domains.

Question No.	Item	Response Options
1	I have read the information above and agree to participate.	Yes / No
2	I am over 18 years of age.	Yes / No
3	I am currently a postgraduate attending primary health care.	Yes / No
4	Email Address (Optional)	[Free text]
Section 1: Demographic Information		
5	How old are you?	[Free text]
6	What is your major?	GP / Family medicine specialist / Family medicine resident / Other
7	What is your current year (experience)?	1-5 yrs / 6-10 yrs / 11-15 yrs / >15 yrs
8	Current relationship status	Single / Married / Committed / Divorced / Engaged / Other
Section 2: Fertility Knowledge and Attitudes		
9	How educated do you feel about fertility issues?	Not at all / Not very / Somewhat / Educated / Very Educated
10	Where did you gain most of your knowledge regarding fertility?	Family / Friends / Doctor / School / Media / Other
11	Do you currently have children?	Yes / No
12	If you answered no to Q11, do you plan to have children?	Yes / No
13	How many children do you want?	[Free text]
14	At what age would you like to have your first/last child?	[Free text]
14a	If you already have a child, at what age did you have your first?	[Free text]
14b	If you have no children or plan not to be a mother, please explain.	[Free text]
15	How confident are you in achieving desired fertility goals?	Not at all / Not confident / Somewhat / Confident / Very confident
16	Potential obstacles to achieving fertility goals?	Pursuit of educational goals - Pursuit of personal interests - Pursuit of career aspirations - Infertility - Financial concerns - Not finding the right partner - Not feeling emotionally ready - More family contact - Other - No obstacles
Section 3: Fertility Beliefs		
17	At what age are women most fertile?	[Free text]
18	At what age is there a slight decrease in fertility?	[Free text]
19	At what age is there a marked decrease in fertility?	[Free text]
20	Chance of pregnancy at ovulation with unprotected sex?	[Percentage]
21a	Chance of pregnancy (25-30 years) over one year unprotected sex?	[Percentage]
21b	Chance of pregnancy (35-40 years) over one year unprotected sex?	[Percentage]
22	Percentage of couples in Saudi Arabia who are involuntarily childless?	[Percentage]
23	Average chance of IVF success after one treatment?	[Percentage]
Section 4: Value of Parenthood and Reactions		
24	How important is it to have children?	0-10 scale
25	Would you undergo IVF if unable to conceive?	0-10 scale
26	Would you adopt if unable to conceive?	0-10 scale
27	Would you choose not to have children?	0-10 scale
Section 5: Motivations for parenthood		
Please rate how important each condition is in your decision to become a parent, using the following scale: Unimportant / Not very important / No opinion / Rather important / Important / Very important		
- Having a supportive partner		
- Feeling mature		
- Being in a stable relationship		
- Having children before being too old		
- Balancing profession and children		
- Completing studies		
- Advancing in profession		
- Having a full-time job		
- Access to childcare		
- Good salary		
- Adequate home size		
- Friends having children		
- Having had time to travel		
